# Study on Photoelectric Properties of Graphene/Molybdenum Disulfide Heterojunction

**DOI:** 10.3390/nano15110787

**Published:** 2025-05-23

**Authors:** Hui Ren, Xing Wei, Jibin Fan

**Affiliations:** School of Materials Science and Engineering, Chang’an University, Xi’an 710064, Chinaweixing@chd.edu.cn (X.W.)

**Keywords:** graphene, heterostructure, dark current, MoS_2_

## Abstract

The zero-bandgap of graphene means that it can achieve a full spectral range response for graphene-based photodetectors. But the zero bandgap of graphene also brings relatively large dark current. To improve this issue and achieve low-cost graphene-based photodetectors, radio frequency (RF) magnetron-sputtered molybdenum disulfide constructed with graphene to form heterojunction was investigated. The results indicated that graphene/molybdenum disulfide heterojunction could provide a Schottky barrier height value of 0.739 eV, which was higher than that of the graphene/Si photodetector. It is beneficial to suppress the generation of the dark current. Different sputtering conditions were also studied. Testing results indicated that for the optimized process, the responsivity, detectivity, and quantum efficiency of graphene/molybdenum disulfide heterojunction photodetectors could reach up to 126 mA/W, 1.21 × 10^11^ Jones, and 34%, respectively. In addition, graphene/molybdenum disulfide heterojunction on flexible PET substrate showed good stability, indicating that graphene/molybdenum disulfide heterojunction also has a good potential application in the field of flexible electronics.

## 1. Introduction

Due to its extremely high electron mobility, zero bandgap, and good optical properties, graphene has been studded as an ideal photosensitive material for optoelectronic devices [1,2,3]. Especially, the feature of graphene’s zero bandgap can achieve a full spectral range response, which reduces the cost of photodetectors because the spectral range of photodetectors is generally determined by the bandgap of the photosensitive material [4]. However, the zero bandgap of graphene also brings relatively large dark current for graphene-based photodetectors due to the generation of electrons and holes. To address this issue, interface engineering such as introducing a passivation layer [5] and using heterostructures [6,7] has been studied to improve the dark current in graphene-based photodetectors. The heterostructure is usually constructed by graphene and other materials to provide a Schottky junction barrier; this has been considered the most suitable method to significantly decrease the dark current.

Molybdenum disulfide (MoS_2_) in Transition metal dichalcogenides materials has a natural tunable band gap and shows strong light absorption capacity [8]. The bandgap of bulk MoS_2_ is 1.2 eV and it increases with a decrease in molybdenum disulfide thickness [9]. A single layer of molybdenum disulfide has a direct band gap of 1.8 eV [9]. MoS_2_/graphene heterostructures integrate the superior light–solid interaction in MoS_2_ and charge mobility in graphene for high-performance optoelectronic devices [10,11]. Molybdenum disulfide/graphene structures have been studied as photodetectors, sensors, and field-effect transistors [12,13]. However, the progress is restricted due to susceptibility of the large-area, low-defect single-layer MoS_2_ in chemical vapor deposition. In order to obtain lower-cost and better-performance graphene-based photodetectors, in this paper, large area molybdenum disulfide films were prepared by a radio frequency (RF) magnetron sputtering method to obtain a molybdenum disulfide/graphene structure, and the properties of molybdenum disulfide/graphene photodetectors were investigated.

## 2. Materials and Methods

**Figure 1:**
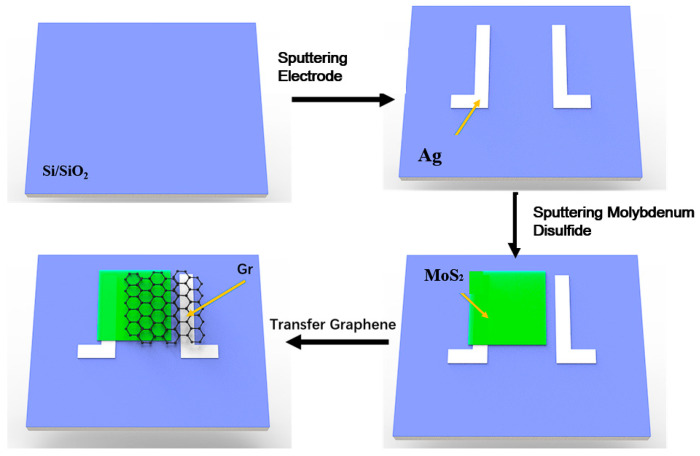
A schematic of the graphene/molybdenum disulfide heterojunction.

Figure 1 illustrates a schematic of a graphene/molybdenum disulfide (Gr/MoS_2_) heterojunction photodetector. Initially, silicon (Si) substrates covered with approximately 300 nm of silicon dioxide (SiO_2_) were cleaned by ultrasonic treatment in acetone and then rinsed with deionized water. Subsequently, the substrates were placed into an RF magnetron sputtering chamber, where patterned silver (Ag) electrodes were deposited using a mask and a high-purity (99.999%, General Research Institute for Nonferrous Metals) silver target. The sputtering power was set at 30 W, and the process lasted for 10 min. After that, patterned MoS_2_ films were deposited by the mask using a high-purity (99.99%, General Research Institute for Nonferrous Metals) MoS_2_ target. The background vacuum during magnetron sputtering was 9.6×10^−^⁴ Pa, and the sputtering pressure was 2.5 Pa. To obtain high-quality MoS_2_ films, experiments on MoS_2_ films under different magnetron sputtering powers (60 W, 80 W, 100 W, and 120 W) were carried out.

Graphene (Gr) was grown on 0.025 mm-thick copper foil (99.8%, Thermo Scientific chemicals, Shanghai, China) by low-pressure chemical vapor deposition (CVD) at 1030 °C, using methane as the carbon source. The copper foils were first cleaned and then folded into a box shape before being placed into the CVD reaction chamber for graphene growth. To transfer the graphene from the copper foil to the substrate, a PMMA solution was spin-coated onto the graphene/copper sample and cured at 150 °C. The graphene/PMMA was then transferred to the substrate with Ag electrodes and MoS_2_ using a bubbling method. The sample was heated again to 120 °C to evaporate water and ensure a strong bond between the graphene and the substrate, followed by the removal of the PMMA using acetone. The photosensitive area of the device was 0.7 cm × 0.7 cm.

Finally, the physical and electrical properties of the materials and devices were tested using X-ray photoelectron spectroscopy (Thermo Scientific, Waltham, MA, USA, K-Alpha), Raman spectroscopy (HORIBA, Kyoto, Japan, HR-800), spectroscopic ellipsometry (VASE, M-2000D Spectroscopic Ellipsometer, J. A. Woollam, Shanghai, China), and a customized photovoltaic testing system (comprising a Keithley 2600 source meter, a probe station, and a laser source with a wavelength range of 400–650 nm).

## 3. Results and Discussion

**Figure 2:**
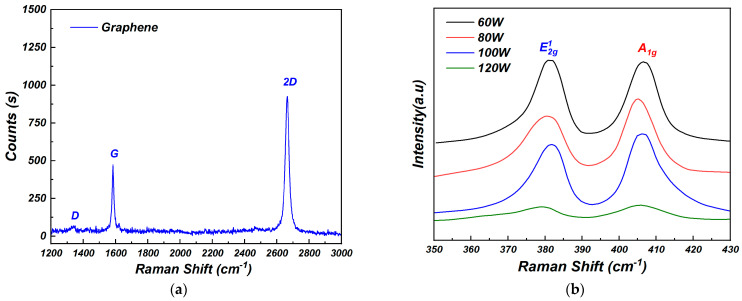
Raman spectra of (**a**) graphene and (**b**) molybdenum disulfide films.

In order to characterize the properties of graphene film, Raman spectroscopy was performed and the results are shown in Figure 2a. The laser wavelength was 532 nm and the power was 100 mW. As shown in Figure 2a, two distinct peaks appeared in the Raman spectrum. They were located at 1587 cm^−1^ and 2682 cm^−1^, which corresponded to the G and 2D peaks of graphene, respectively [14]. Almost no peak was observed at 1350 cm^−1^, indicating that the graphene had a negligible level of defects and good quality [14]. Using the peak intensities of G and 2D, I_2D_/I_G_ > 2 could be obtained. The results indicate that the graphene obtained by the CVD method was a high-quality monolayer structure.

To find out the RF magnetron sputtering process effects on the properties of molybdenum disulfide films, different sputtering powers (60 W, 80 W, 100 W, and 120 W) under 15 min sputtering time were studied. Raman spectroscopy measurements were used to analyze the prepared films, as shown in Figure 2b. The incident laser was selected as 532 nm and the laser power was 100 mW. As shown in Figure 2b, two obvious diffraction peaks appeared in Raman spectra of the molybdenum disulfide prepared at different sputtering powers. These two diffraction peaks corresponded to the Mo-S bond perpendicular to the interlayer-direction out-of-plane relative vibration mode $E_{2g}^{1}$ and the Mo-S bond along the in-plane relative vibration mode A_1g_ [15], which are the Raman characteristic diffraction peaks of 2H-MoS_2_. When the sputtering power increased from 60 W to 120 W, the peak intensity of A_1g_ and $E_{2g}^{1}$ increased. This may be attributed to the molybdenum and sulfur atoms not being fully excited to the substrate surface under the low-sputtering-power condition [16]. High sputtering power resulted in the incomplete combination reaction of molybdenum and sulfur atoms, which led to the degradation of the film. Meanwhile, the distance between the diffraction peaks of $E_{2g}^{1}$ and A_1g_ decreased, especially when the sputtering power reached 100 W. The results indicate that choosing the sputtering power of 100 W was beneficial to obtain high-quality molybdenum disulfide film. For the 100 W-sputtering-power sample, $E_{2g}^{1}$ and A_1g_ was located at 382.8 cm^−1^ and 409.5 cm^−1^, respectively. The difference between these two peaks was 26.7 cm^−1^, which indicated that the MoS_2_ film was between five and seven layers [17].

**Figure 3:**
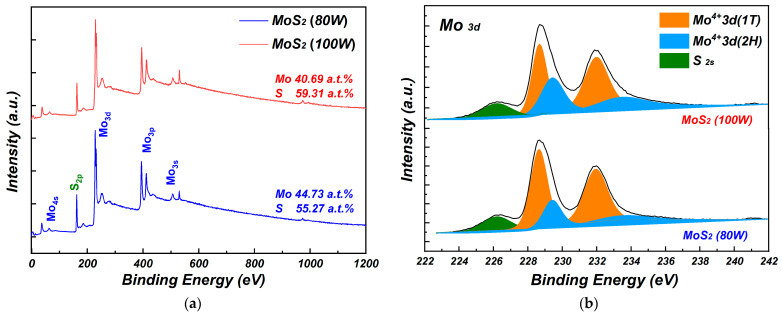
(**a**) Full spectra and (**b**) Mo 3d XPS spectra of molybdenum disulfide.

The properties of molybdenum disulfide film deposited at 100 W sputtering power was further analyzed by the XPS, as shown in Figure 3. All spectra were calibrated with a C_1s_ peak of 284.8 eV to eliminate the charge effect. As shown in Figure 3a, except for the elemental peaks introduced by the substrate, the main peaks were attributed to Mo and S. When the sputtering power increased from 80 W to 100 W, the ratio of Mo atoms decreased from 44.73 a.t.% to 40.69 a.t.% and the ratio of S atoms increased from 55.27 a.t.% to 59.31 a.t.%. The value of Mo/S changed from 1/1.23 to 1/1.45, which was closer to the standard stoichiometric ratio of 1/2. This indicates that the molybdenum disulfide prepared by magnetron sputtering was composed of molybdenum-rich sulfur deficiency, which is consistent with the results of molybdenum disulfide prepared by magnetron sputtering reported in the literature [16]. Compared to the molybdenum disulfide film deposited at 80 W sputtering power, the stoichiometric ratios of Mo and S atoms were closer to the ideal values. This may be attributed to the fact that higher sputtering power could make the Mo and S atoms be in higher excited states with higher reactivity, which was beneficial for reaction and bonding. Figure 3b shows the XPS peak fitting results of Mo_3d_ and S_2s_. The results indicate that Mo_3d_ was a typical double-peak, and its binding energies were located at 228.5 eV, 229.4 eV, 231.9 eV, and 233.3 eV, respectively [16]. The Mo^6+^_3d_ peak was not found, indicating that the molybdenum disulfide film had no oxidation [18]. The Mo_3d_ peaks at low binding energy belonged to Mo^4+^ in metallic 1T-MoS_2_, while the peaks at high binding energy belonged to Mo^4+^ in semiconducting 2H-MoS_2_ [16]. When the sputtering power was increased from 80 W to 100 W, an increase in the component of Mo_3d_ in 2H-MoS_2_ could be obtained from the area of the fitting peaks. All results indicate that molybdenum disulfide sputtered at a sputtering power of 100 W showed a suitable Mo/S ratio and more components of the semiconductor phase.

**Figure 4:**
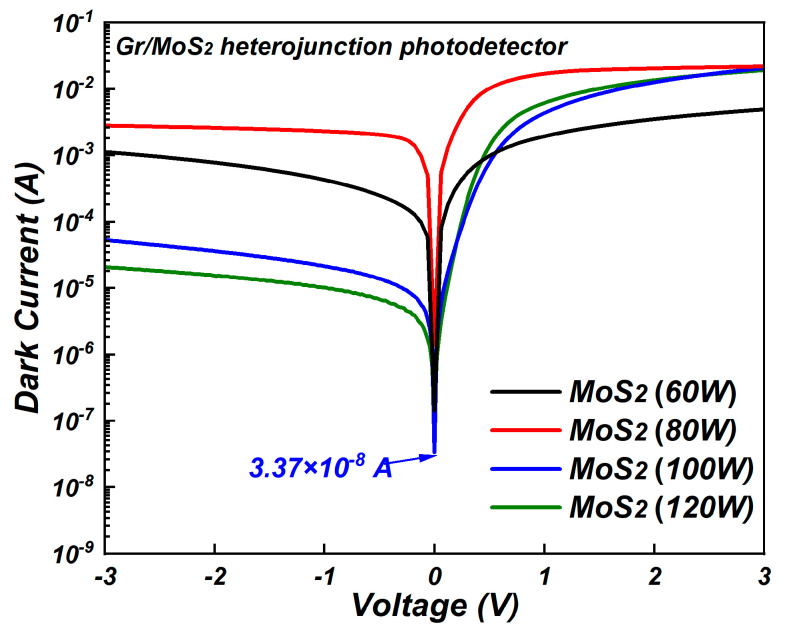
I–V results of graphene/molybdenum disulfide heterojunction photodetectors under dark conditions.

To evaluate the dark current of the graphene/molybdenum disulfide heterojunction, the graphene/molybdenum disulfide heterojunction photodetectors were produced and the I–V measurements were tested under dark conditions. As shown in Figure 4, except for the molybdenum disulfide at a sputtering power of 80 W, the dark current of the graphene/molybdenum disulfide heterojunction photodetectors exhibited a decreasing trend as the sputtering power increased from 60 W to 120 W. At a bias voltage of 0 V, the dark current of graphene/molybdenum disulfide heterojunction photodetectors prepared at the sputtering powers of 60 W, 80 W, 100 W, and 120 W was 1.14 × 10^−7^ A, 1.33 × 10^−6^ A, 3.37 × 10^−8^ A, and 1.08 × 10^−7^ A, respectively. The dark current reached a minimum value of 3.37 × 10^−8^ A for molybdenum disulfide at a sputtering power of 100 W. This is comparable with the dark current value of photodetectors based on mechanically exfoliated MoS_2_ on graphene [18]. Furthermore, it is an order of magnitude lower than the dark current value of ~10^−7^ A for graphene/Si photodetectors [19]. The results indicate that using the magnetron-sputtered molybdenum disulfide to form the graphene/molybdenum disulfide heterojunction could decrease the dark current of the graphene photodetectors based on the advantages of large area and low cost.

**Figure 5:**
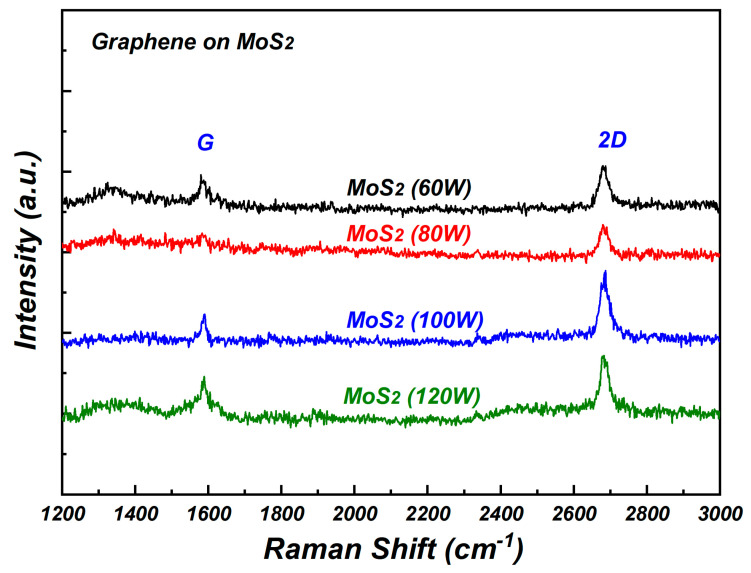
The Raman spectra of graphene on MoS_2_ with different sputtering conditions.

In order to find out the changes in dark current, the Raman spectra of graphene on MoS_2_ were measured and the results are shown in Figure 5. The G peak and 2D peak could be found in the Raman spectra of graphene on MoS_2_ with different sputtering conditions. The presence of G and 2D peaks prove the existence of graphene on MoS_2_. For molybdenum disulfide sputtered at a sputtering power of 80 W, the weak response of G peak was observed compared with in other sputtering conditions. The G-band represents the planar configuration *sp*^2^ bonded carbon that constitutes graphene and it is very sensitive to the effects of stress. The decrease in the G peak may have been caused by the adsorption of atoms on graphene surfaces disrupted the symmetry of the graphene’s stacking structure [14]. The degradation of graphene occurred when the graphene was transferred to molybdenum disulfide substrate sputtered at a sputtering power of 80 W, leading to the largest dark current for molybdenum disulfide at a sputtering power of 80 W in Figure 4.

**Figure 6:**
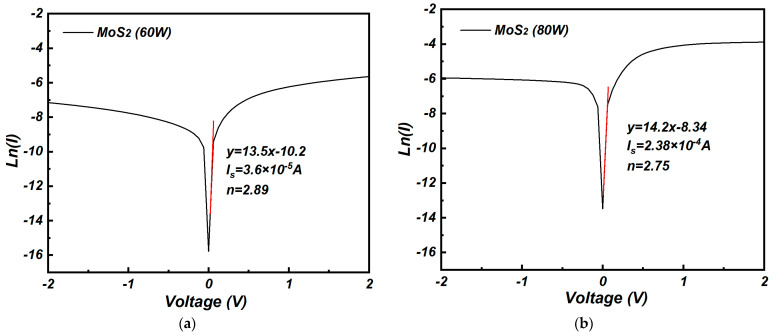

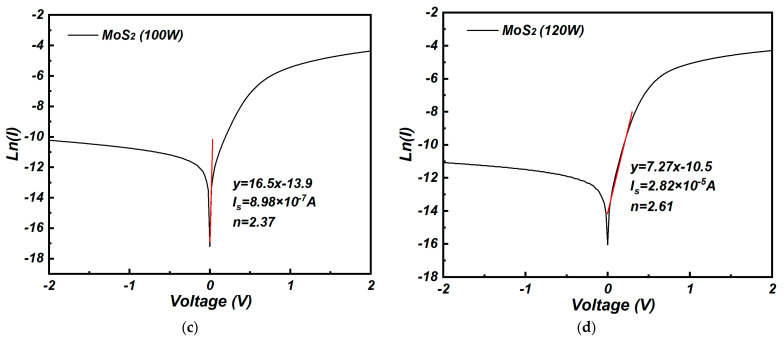
ln(I)-V fitting curves of the graphene/MoS_2_ photodetectors: (**a**) 60 W, (**b**) 80 W, (**c**) 100 W, (**d**) 120 W.

The Schottky barrier height values of graphene/molybdenum disulfide heterojunction photodetectors were calculated using the following formulas [20,21]:

(1) $$\varphi_{b}=\frac{KT}{q}\ln\left(\frac{A_{eff}A^{*}T^{2}}{I_{s}}\right)$$

(2) $$\ln\left(I\right)=\ln\left(I_{s}\right)+\frac{qV}{nKT}$$

where $\varphi_{b}$ represents the Schottky barrier height, *A_eff_* is the effective working area of the photodetector, A* is the Richardson constant (0.70 × 10^−6^ A cm^−2^ K^−2^ for MoS_2_), *I_s_* is the reverse saturation current, *q* is the elementary charge with a value of 1.602 × 10^−19^ C, K is the Boltzmann constant (1.38 × 10^−23^ J/K), T is the absolute temperature, and *n* is the ideal factor of the device. The fitting results of I-V curves in Figure 4 are shown in Figure 6.

As shown in Table 1, the Schottky barrier height ($\varphi_{b}$) of the graphene/molybdenum disulfide heterojunction prepared at the sputtering powers of 60 W, 80 W, 100 W, and 120 W was 0.644 eV, 0.596 eV, 0.739 eV, and 0.721 eV, respectively. For molybdenum disulfide at a sputtering power of 100 W, the Schottky barrier height of the graphene/molybdenum disulfide heterojunction reached maximum value of 0.739 eV. A higher Schottky barrier height was beneficial to decrease the transportation of carriers to form the dark current. Meanwhile, the ideal factor was calculated to be 2.3. According to PN junction theory, when the ideal factor approaches or equals 2, it indicates that the current transport mechanism within the device has shifted from diffusion current to recombination current [22]. The excessive recombination current was attributed to a high density of defects at the interface, which increased carrier recombination. This may have been related to defects present on the surface of the fabricated graphene and the numerous dangling bonds on the sputtered MoS_2_ surface, leading to significant recombination current at the interface.

**Figure 7:**
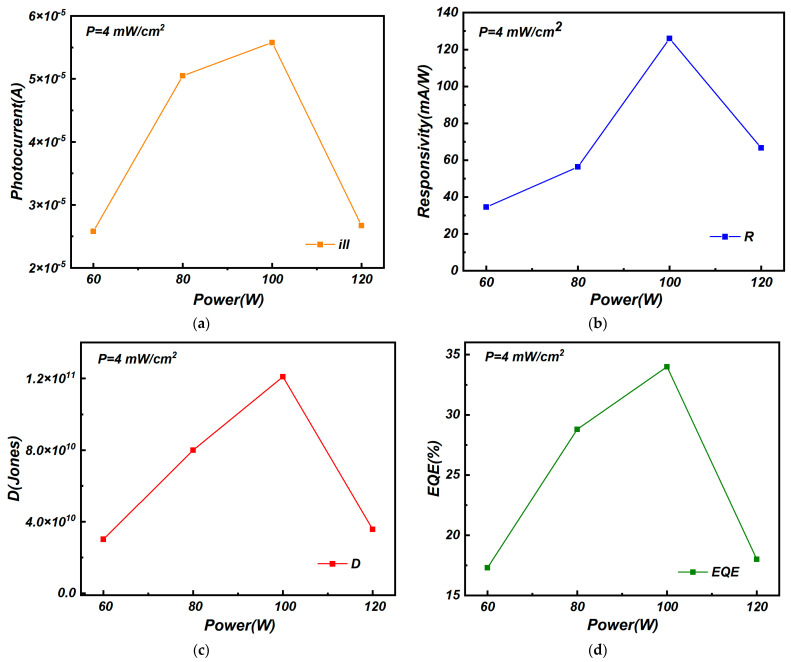
(**a**) Photocurrent, (**b**) responsivity, (**c**) detectivity, and (**d**) quantum efficiency of Gr/MoS_2_ heterojunction photodetectors at different sputtering powers.

To further evaluate the performances of the graphene/molybdenum disulfide heterojunction photodetectors under illumination conditions, I-V measurements were carried out and the calculated parameters of the graphene/MoS_2_ heterojunction photodetectors are shown in Figure 7. For illumination conditions, an incident light wavelength of 460 nm with an incident power of 4 mW/cm^2^ was used. As shown in Figure 7a, the photocurrent of graphene/molybdenum disulfide photodetectors increased firstly as the sputtering power increased from 60 W to 100 W and then decreased. At the sputtering power of 100 W, the photocurrent of the graphene/molybdenum disulfide photodetector reached the maximum value of 5.58 × 10^−5^ A. The main reason for this phenomenon was that the molybdenum and sulfur atoms could not be fully excited to the substrate surface in the low-sputtering-power condition, while high sputtering power resulted in the incomplete combination reaction of molybdenum and sulfur atoms.

As shown in Figure 7b, the responsivity of the graphene/molybdenum disulfide photodetectors also changed with the sputtering power. It was consistent with the variation in the photocurrent. For molybdenum disulfide in the 100 W-sputtering-power condition, the responsivity of the graphene/molybdenum disulfide photodetector reached the maximum value of 126 mA/W. Meanwhile, the measured detectivity values of the graphene/molybdenum disulfide photodetectors, shown in Figure 7c, and the quantum efficiency, shown in Figure 7d, were 1.21 × 10^11^ Jones and 34%, respectively. They also reached maximum values under the 100 W-sputtering-power condition. The results suggest that, owing to the reduction in dark current and high quality of molybdenum disulfide in the 100 W-sputtering-power condition, the graphene/molybdenum disulfide photodetector could achieve the best photodetection properties.

**Figure 8:**
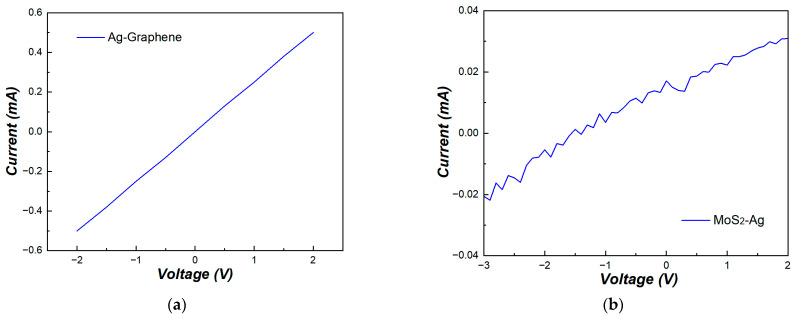
Current–voltage characteristics of silver electrode contact with (**a**) graphene and (**b**) molybdenum disulfide.

In addition, the Ag electrode-and-graphene and Ag electrode-and-molybdenum disulfide contact characteristics were also studied. The current–voltage result of the Ag electrode-and-graphene contact is shown in Figure 8a. The linear relationship observed in the curves indicates that the Ag electrodes formed ohmic contact with the graphene, rather than Schottky contact. In Figure 8b, the contact of the Ag electrode and molybdenum disulfide shows a linear-like trend with a contact resistance of approximately 368 mΩ. This may be attributed to the amount of sulfur vacancies in the molybdenum disulfide, which could successfully dope the contact regions by introducing sulfur vacancies [23].

**Figure 9:**
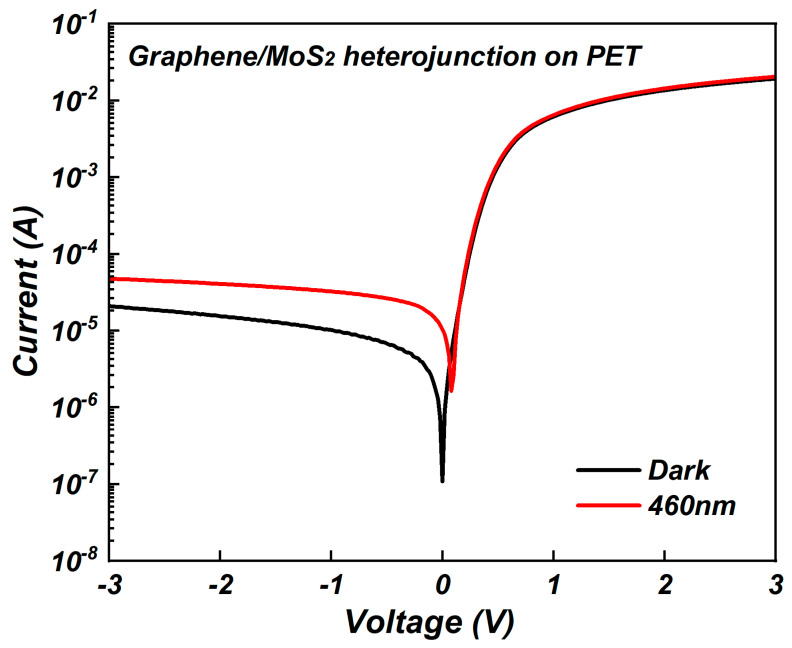
I–V results of graphene/molybdenum disulfide heterojunction on PET.

The application of graphene/molybdenum disulfide heterojunctions in flexible photodetectors were also investigated. Molybdenum disulfide was deposited on polyethylene terephthalate (PET) substrate using a sputtering power of 100 W to construct the graphene/molybdenum disulfide heterojunction. The schematic of the graphene/molybdenum disulfide heterojunction on PET is the same as that in Figure 1. Under illumination, incident light with a wavelength of 460 nm and a power of 4 mW/cm^2^ was used. The I–V results are shown in Figure 9. At a bias voltage of 0 V, the dark current of the graphene/molybdenum disulfide heterojunction photodetector was 6.54 × 10^−5^ A, which was higher than the dark current of 3.37 × 10^−8^ A on silicon dioxide substrate under the same conditions. This may have been due to the flexible nature of PET leading to more defects within the material, increasing carrier recombination and thus raising the dark current. At a bias voltage of 0 V, when the incident light wavelength was 460 nm, the net current, responsivity, and detectivity of the graphene/molybdenum disulfide heterojunction photodetector on the flexible PET substrate were 2.53 × 10^−5^ A, 6.32 mA/W, and 2.85 × 10^9^ Jones, respectively. Despite the higher dark current on the PET substrate, the response photocurrent under illumination still indicates the potential application of this heterojunction in flexible photodetectors.

**Figure 10:**
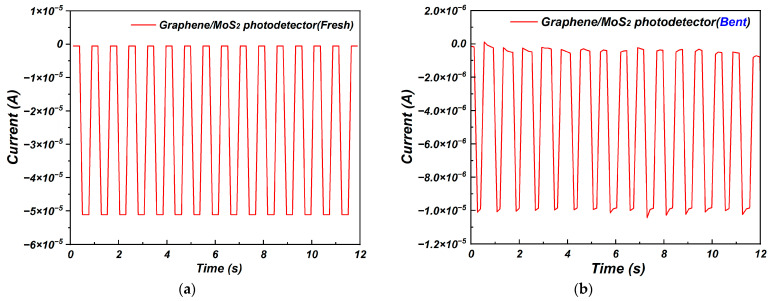
I–T curves of graphene/molybdenum disulfide heterojunction photodetectors on flexible PET substrate: (**a**) fresh and (**b**) after bending 30° and 50 times.

The stability of graphene/molybdenum disulfide photodetectors on flexible PET substrates was also assessed. An I–T test was carried out to evaluate whether the graphene/molybdenum disulfide heterojunction photodetectors on PET substrate could work stably after bending 30° and 50 times, as shown in Figure 10. The graphene/molybdenum disulfide photodetectors on flexible PET substrate showed good stability when the device was not bent. After bending 30° and 50 times, graphene/molybdenum disulfide photodetectors on flexible PET substrate also showed stability, although the light response showed negligible variation after bending. This may have been caused by the change in the microstructure in the material due to frequent bending, which may have caused fatigue effects and reduced the performance of the material.

## 4. Conclusions

In this study, experiments on a graphene/molybdenum disulfide heterojunction were fabricated to improve the dark current of graphene-based photodetectors. The Raman results indicate that the CVD graphene had low defects and was single-layered. The magnetron-sputtered molybdenum disulfide was affected by the sputtering power and few-layered molybdenum disulfide with good properties could be obtained under the 100 W sputtering power. The XPS results indicate that the molybdenum disulfide prepared by magnetron sputtering was molybdenum-rich sulfur-deficient molybdenum disulfide film. Because of this, the graphene/molybdenum heterojunction could provide a higher Schottky barrier height value. As a result, the responsivity of the graphene/molybdenum disulfide heterojunction photodetectors could reach 126 mA/W, the detectivity was 1.21 × 10^11^ Jones, and the quantum efficiency was 34%. In addition, graphene/molybdenum heterojunction also showed stability on flexible PET substrates, demonstrating its potential application in the field of flexible electronics.

## Figures and Tables

**Table 1:** Device performance parameters of MoS_2_ at different sputtering powers.

Power	n	Is (A)	$\varphi_{b}$ (eV)
60 W	2.89	3.6 × 10^−5^	0.644
80 W	2.75	2.38 × 10^−4^	0.596
100 W	2.37	8.98 × 10^−7^	0.739
120 W	2.61	2.84 × 10^−5^	0.721

## Data Availability

No new data were created or analyzed in this study.
